# The Long-Term Influence of Puppy Acquisition on Physical Activity: Results of a 3-Year, Longitudinal, Pilot Study

**DOI:** 10.3390/healthcare10091687

**Published:** 2022-09-04

**Authors:** Benedikt Hielscher-Zdzieblik, Udo Gansloßer, James Serpell, Ingo Froboese

**Affiliations:** 1 Institute of Movement Therapy and Movement-Oriented Prevention and Rehabilitation, German Sport University Cologne, 50933 Cologne, Germany; 2 Institute of Zoology and Evolutionary Research, Friedrich Schiller University Jena, 07743 Jena, Germany; 3 Department of Clinical Sciences & Advanced Medicine, School of Veterinary Medicine, University of Pennsylvania, Philadelphia, PA 19104, USA

**Keywords:** dog-related physical activity, dog acquisition, longitudinal, dog walking, puppy acquisition, dog-related exercise, COVID-19 pandemic

## Abstract

Dog ownership has been associated with increased levels of physical activity (PA), including increases in total PA and walking time in some but not in all studies. These earlier studies did not consider puppy acquisition, in particular, and were limited to a maximum of 10 months duration. The purpose of the current pilot study was, therefore, to analyze long-term changes in participants’ PA behavior after puppy acquisition. Participants who acquired a puppy from one of nine preselected breeds differing in size and energy level were included. PA was assessed at baseline and 6, 12, 24 and 36 months after puppy acquisition using an online questionnaire. Participants, who were initially included, did not differ in their PA at baseline (*n* = 38). In the group that completed the trial (*n* = 11) total PA, leisure time walking, total dog-related PA, and total dog walking increased within the first two years and decreased while remaining above baseline values during the last year, coinciding with the COVID-19 pandemic. Non-dog-related PA did not change over time. We conclude that the acquisition of a puppy increases PA and thus, could have a long-lasting positive effect on physical health.

## 1. Introduction

Physical activity (PA) is associated with decreased mortality [1,2,3] and a lower rate of several chronic diseases [4,5]. The World Health Organization (WHO) recommends that people be active for at least 150 min per week at a moderate intensity [6]. These guidelines are more often achieved by dog owners than non-dog owners [7,8,9,10,11].

Several studies have shown that dog ownership is associated with increased levels of PA in Australia [7], Canada [12], the Czech Republic [13,14], Germany [15], Japan [11], South Korea [16] and the United Kingdom [8,10,17,18,19,20].

Therefore, it is plausible that the incidence of several diseases including acute myocardial infarction, and cardiovascular and all-cause mortality is lower amongst dog owners, as shown by Mubanga et al. [21]. However, according to the findings of two meta-analyses, it is still unclear whether owning a dog is associated with reduced mortality [22,23]. While Kramer et al. [22] indicate that there is a negative association, Bauman et al. [23] could only confirm this effect for individuals with prior cardiovascular disease.

There are some dog-related factors that are associated with dog walking (see e.g., [24,25,26,27,28,29,30,31,32,33]). Several studies show that the age of the dog is negatively correlated with the PA of dog owners [24,25,26,27]. However, puppies are reported to sleep more during the day than young adult dogs [29] which might in turn influence the PA of the owners negatively. Furthermore, the energy level [26,30] and the size of the dog or dog breed [30,31,32,34,35] are positively associated with the PA of dog owners. Even though there are other factors that are reported to be associated with dog walking (e.g., the age of the owner [25,33] or the type of residence [36]), this study focuses exclusively on the mentioned dog-specific factors (age, size and energy level of the dog).

In cross-sectional studies, only correlations, but no causal conclusions can be drawn [37]. Therefore, longitudinal studies are very important. Nevertheless, longitudinal studies on PA behavior in dog owners are rare. The first study in this research field demonstrates that acquiring a dog led to an increase in dog walking [38]. However, an Australian study concluded that after the acquisition of the dog, walking replaces other PAs and that the total level of PA remains unchanged [39]. Powell et al. [40] showed an increase in steps three months after acquiring a dog, but eight months after the acquisition, daily steps decreased again, though they were still higher than at baseline. Potter et al. [41] assessed an increase in daily steps six weeks after dog acquisition. However, daily steps decreased after 12 weeks in comparison to the six-week measurement but were still increased compared to baseline [41].

To the best of our knowledge, all of the longitudinal studies focused on a relatively short period of time with a maximum observation period of 10 months and they did not differentiate between dog breeds, sizes and energy levels of the dogs [38,39,40,41]. Additionally, none of these studies focused on the effect of puppy acquisition on PA in their owners [38,39,40,41].

There is some evidence that certain exercises, as well as overexercising might be harmful to young dogs [42,43]. The Swiss Veterinary Association for Behavioral Medicine and the American Veterinary Medical Association recommend only reduced PA for puppies, with a stepwise increase depending on the growth pattern of the individual dog [44,45]. It is, therefore, conceivable that the acquisition of a puppy does not have an immediate effect on dog owners’ PA, but that this effect is dependent on the growth process of the animals. However, since the growth process differs by the size of the adult dog—larger dogs take longer to mature [46]—it is also possible that the effect of puppy acquisition on PA in their owners differs between dogs of different sizes.

The main purpose of the study was to determine whether the acquisition of a puppy has an influence on long-term PA in dog owners. Furthermore, the study aimed to find differences in PA and changes in PA over time in owners of different dog breeds.

## 2. Materials and Methods

### 2.1. Participants

People who were going to acquire a pure-bred puppy from one of nine different dog breeds from a dog breeder were eligible to participate in the study. They had to be at least 18 years old and be able to understand German. To ensure that the PA of previous dog owners resembled that of non-dog owners, it was initially required that prospective dog owners must not have owned a dog in the previous three months prior to acquiring the new puppy. This requirement was withdrawn in the course of the investigation due to the low rate of participant recruitment.

Dog breeders of the selected breeds were contacted via phone or e-mail. They were contacted if they had registered a litter of puppies of one of the selected breeds (as described in the procedure below) on the publicly available website of a breed association between 1 February 2017 and 31 March 2018. Overall, 996 litters were documented (*n* = 44–246 per breed).

The first author explained the procedure and the purpose of the study. The breeders also received two letters via e-mail that explained the procedure. One was for the breeders and one was addressed to possible study participants. The latter contained information about the study and the contact information for the first author, who could be contacted via e-mail or phone in case of any questions or concerns about the study or procedure. Due to data protection requirements, participants could not be contacted directly by the first author. Thus, the breeders were asked to pass on the information to possible participants.

**Figure 1:**
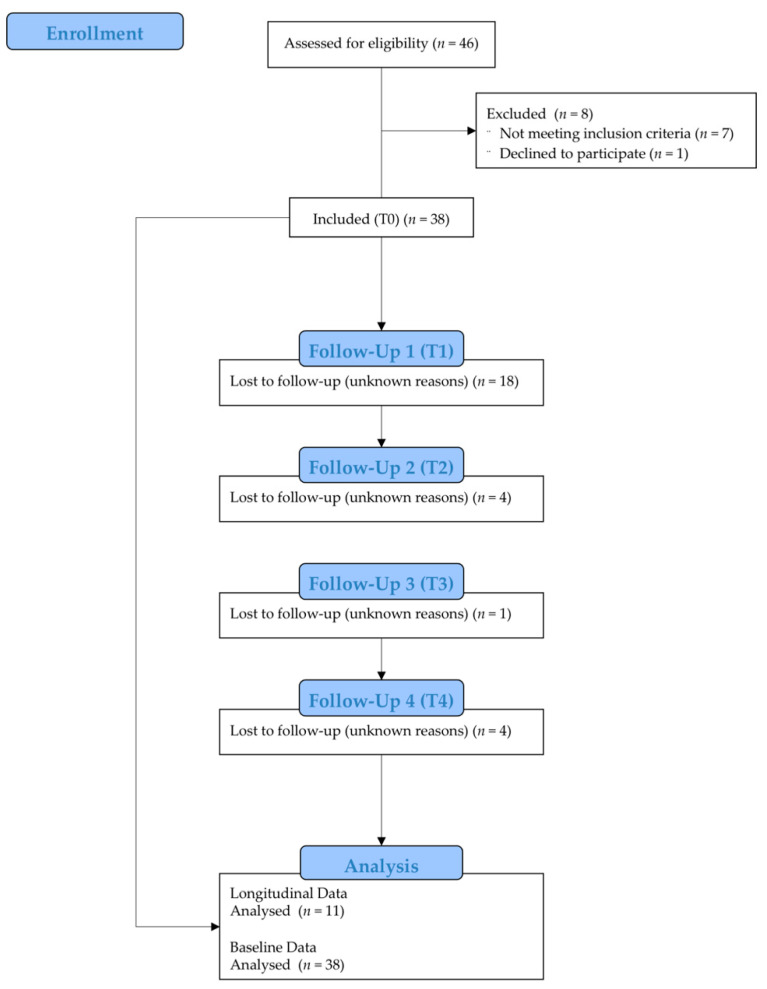
Flow chart of participants over the course of the study.

Overall, 46 participants were assessed for eligibility. One participant was excluded because no informed consent was given. Seven participants did not meet the inclusion criteria and were, therefore, excluded. The course of the study is shown in Figure 1.

A total of 38 dog owners met the inclusion criteria and participated in the study (Figure 1). Eleven participants completed the trial and were included in the main statistical analysis. Thus, the dropout rate was 71.1%. Dropouts were due to voluntary withdrawal from further participation after the initial examination.

### 2.2. Questionnaires

Participants completed an online questionnaire at baseline (T0), 6 (T1), 12 (T2), 24 (T3) and 36 (T4) months after puppy acquisition. Informed consent was given actively at the beginning of the questionnaire. Without this, it was not possible to complete the questionnaire any further. Then participants were required to give their e-mail addresses and a personal code. Further, they were asked which breed of dog they were going to acquire.

At baseline, participants were also asked to report sociodemographic data (Table 1).

After giving this information, participants completed the German version of the Physical Activity, Exercise and Sport Questionnaire (Bewegungs- and Sportaktivitätsfragebogen [BSA-F]), a validated questionnaire to assess physical and sports activity [47]. The BSA-F asks for PA that was performed four weeks prior to evaluation [47]. It is a subjective, self-reported questionnaire and was completed at T0, T1, T2, T3 and T4. The BSA-F measures work-related PA as an index value. This value was not used in this study. In the BSA-F, total PA is divided into activities of daily living (ADL) and exercise-related activities, with leisure time walking being one item of the ADL-section [47]. The BSA-F surveys PA by asking about the frequency and duration of certain ADLs. For exercise-related questions, the type of PA can be self-reported in an open text field. The BSA-F was validated by Fuchs et al. [47]. The results of the BSA-F correlate with the anaerobic threshold, increased power at the anaerobic threshold and maximum oxygen consumption [47]. Additionally, the participants were asked about dog-related PA and dog walking, in particular, using the same wording as the BSA-F. This technique was also used in previous studies in Germany [15,48,49].

### 2.3. Procedure

The study was designed as a prospective, open pilot trial conducted at the German Sport University Cologne, Germany. The breeds were selected based on their height at the withers and their energy level. To ensure a sufficient sample size within each breed group an average of at least 500 puppies per dog breed per year should have been born in Germany within the last five years. The average number of puppies born per year was calculated using the official statistics of the German Kennel Organization (VDH) from 2010 to 2014 [50]. For the body size, the details of the breed standard of the Fédération Cynologique International (FCI) were taken into account. Breeds were categorized by the height of the withers as follows:

- Small: <40 cm
- Medium: 40–59 cm
- Large: ≥60 cm

The largest value given in the breed standard was crucial in each case. In addition, the attributed energy level was used to select the dog breeds. The following dog breeds were selected in this study:

- Small:
  - Cavalier King Charles Spaniel (CKCS) [51] (low energy)
  - West Highland White Terrier (WHWT) [52] (medium energy)
  - Parson Russel Terrier (PRT) [53] (high energy)
- Medium:
  - Whippet (WHI) [54] (low energy)
  - Labrador Retriever (LAB) [55] (medium energy)
  - Border Collie (BC) [56] (high energy)
- Large:
  - Bernese Mountain Dog (BMD) [57] (low energy)
  - Rottweiler (ROT) [58] (medium energy)
  - Belgian Shepherd Dog (BSD) [59] (high energy)

To confirm that the dog breeds within each size category differ in their energy level, the energy level of each breed was evaluated using data from the Canine Behavioral Assessment and Research Questionnaire (C-BARQ) project as previously described [60]. The C-BARQ project, based at the University of Pennsylvania since 2005, includes behavioral analysis data from over 70,000 individual dogs from over 300 breeds [60]. The C-BARQ is a validated instrument for evaluating individual behavior and temperament in dogs [61]. It contains 14 dimensions of which Energy is one. It is described by two items which are described as “playful, puppyish, boisterous” and “active, energetic, always on the go” [62]. Energy was used to differentiate between the level of activity of the dog breeds in each size category. This dimension has been shown to differ between dog breeds [62] and breed groups [63]. Significant differences between the dog breed groups exist within each size category used in this study, as previously shown by Hielscher-Zdzieblik et al. [48].

For the baseline, participants had to complete the online questionnaire before they acquired a dog or within ten days after acquisition between 1 June 2017 and 31 May 2018. Dog owners that completed the questionnaire later were excluded from the analysis.

Participants were asked at baseline to specify the date on which they were going to acquire the dog from the breeder. This date was used for calculating the date to contact participants for the retests (T1–T4). They were invited at each timepoint with a personalized e-mail to complete the questionnaire again. They received two reminder e-mails ten days apart. If participants did not answer the questionnaire for a retest, this was considered as a voluntary withdrawal from further participation. Hence, they did not receive any further reminders at the subsequent time points. The questionnaire did not ask participants for their reasons for withdrawing from the study.

### 2.4. Statistical Analysis

All metric data are presented as mean (*M*) ± standard deviation (*SD*) The median (*Mdn*) is shown with interquartile ranges (IQR) for longitudinal data. Categorical data are displayed in absolute values and percentages.

Due to the small sample size, only non-parametric tests were used. Differences in categorical variables were analyzed using χ^2^-tests. Mann–Whitney-U-tests were used to compare data between two groups. χ^2^-tests and Mann–Whitney-U-tests were only used to compare baseline data.

At baseline, the breeds were grouped by size and energy level. Groups were analyzed for differences in total PA und walking behavior using the Kruskal–Wallis-test. For the Kruskal–Wallis-test statistics, the *H*-value is displayed.

Differences within the whole cohort during the course of intervention were analyzed using a Friedman-test.

Effect sizes were calculated using Microsoft Office Excel for Mac, Version 16.43. Epsilon-squared (*E*^2^) was used for Kruskal–Wallis tests and Kendall’s *W* was used for the Friedman tests as suggested by Tomczak and Tomczak [64]. Tomczak and Tomczak state that the value for *E*^2^ and Kendall’s *W* can range from 0 to 1, where 0 represents no relationship and 1 represents a perfect relationship [64].

IBM SPSS Statistics for Mac, Version 27.0 (Armonk, NY, USA) was used in all statistical analyses. The level of statistical significance was set at α = 0.05. Boxplots were created using Microsoft Office Excel for Mac, Version 16.43.

Since the present investigation was considered a pilot trial, endpoints are regarded equivalently and correction of the level of significance was not performed.

## 3. Results

The descriptive analysis of the baseline socioeconomic data of participants is presented in Table 2. In the total sample, the mean age was 44.9 ± 10.8 years. In the subset of participants who completed the study, the mean age was 47.9 ± 11.7 years. The mean BMI in the total sample was 26.9 ± 6.9 kg/m^2^ and 27.4 ± 7.8 kg/m^2^ in the completing population.

Overall, in the total sample, seven participants (18.4%) had never owned a dog before acquiring a new puppy. Thirteen participants (34.2%) reported that they had owned a dog before but did not at the beginning of the study. The average duration since they owned their last dog was 7.3 ± 8.3 years. Eighteen participants (47.4%) owned a dog at the beginning of the study. Of these, eight reported owning one dog, five reported having two dogs, two reported having three, and the same number reported having four dogs. Finally, one participant reported owning five dogs.

In the completing population, two participants (18.2%) had never owned a dog before. Three participants (27.3%) indicated that they had owned a dog in the past but did not own a dog at baseline. The average time since owning the previous dog was 3.4 ± 4.8 years. Overall, six (54.5%) of the completing owners reported owning a dog at baseline. Of these, three reported owning one dog, two owned two dogs and one owned three dogs at baseline.

At baseline, completing participants did not differ from dropouts in age, BMI, gender, smoking status, relationship status, size of hometown, garden ownership, employment status, income, current dog ownership, state of residence, educational status, leisure time walking, total PA, dog-walking or dog related PA.

In the initially included population (*n* = 38), there were no significant baseline differences in respect to total PA in h/week (Kruskal–Wallis *H*(2) = 0.07, *p* = 0.968, *E*^2^ = 0.01) or leisure time walking in h/week (Kruskal–Wallis *H*(2) = 0.07, *p* = 0.966, *E*^2^ < 0.01) when comparing owners of dog breeds of different in sizes.

Taking the energy level of the selected dog breeds into account, there were no baseline differences between the owners in total PA in h/week (Kruskal–Wallis *H*(2) = 0.60, *p* = 0.742, *E*^2^ = 0.02) or in leisure time walking in h/week (Kruskal–Wallis *H*(2) = 3.46, *p* = 0.178, *E*^2^ = 0.09).

**Figure 2:**
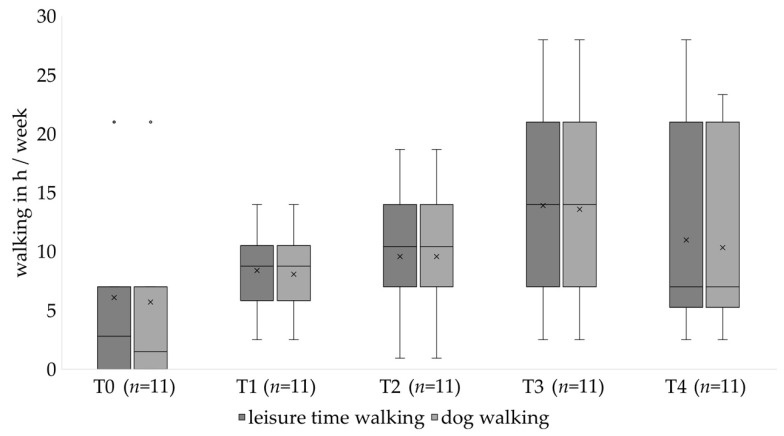
Change of walking behavior over the course of the study. X represents the mean. ◦ represents an outlier.

Analysis of the completing population shows statistically significant changes over time in leisure time walking (*p* = 0.018) and dog walking (0.020) (Table 3). Completing participants exhibited a steady increase in leisure time and dog walking from T0 to T3, with a decrease at T4 (Table 3, Figure 2).

**Figure 3:**
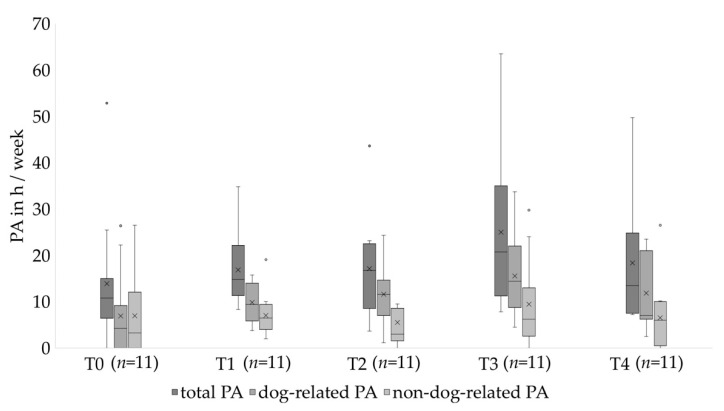
Change of total, dog-related and non-dog-related PA over the course of the study. X represents the mean. ◦ represents an outlier.

Furthermore, there was a statistically significant change over time in total PA (*p* = 0.024) and dog-related PA (*p* = 0.039) in the completing population (Table 3). Total and dog-related PA increased from T0 until T3 and decreased at T4 (Table 3, Figure 3).

Among completers, no significant change over time was found in non-dog-related PA over time (*p* = 0.419) (Table 3, Figure 3).

## 4. Discussion

The main finding of the present study is that there was an increase in total PA, dog-related PA, leisure time walking and dog walking, but not in non-dog related PA after puppy acquisition. This change was most prevalent after 24 months but decreased after 36 months. At baseline, participants who differed according to the size or energy level of their selected dog breeds did not differ in total PA and leisure time walking.

It is plausible that dog-related PA is a substantial contributor to the increase in overall PA. Westgarth et al. [65] and Hielscher et al. [66] state that many dog owners acquire a dog for exercise. Therefore, it is logical that purchasing a dog could increase PA in dog owners.

Some longitudinal studies with similar study designs have been performed before. Serpell [38] demonstrated a relevant increase in walking behavior in the UK. Potter et al. [41] showed moderate increases in PA in the USA. Powell et al. [40] demonstrated an increase in walking minutes, in daily steps and in walking bouts of at least ten minutes at three and eight months after dog acquisition in Australian adults. Their results are not significant, which they attribute to a lack of statistical power [40]. Another Australian study showed significant increases in dog walking, but not total PA [39]. The authors concluded that dog walking replaced other PA in this population [39]. The current results are in line with the findings of Serpell [38] and show that all PA measures, except non-dog-related PA changed over time. However, a comparison between the studies has to be regarded with caution, since in earlier studies the participants most likely acquired adult dogs from shelters [38] or rescue organizations [41] or did not specify how the dogs were acquired [39,40]. Dogs that are acquired in different ways have different character traits (including energy level) [67,68,69]. For example, dogs acquired from pet shops show increased aggression if compared to puppies acquired from noncommercial breeders [67,68]. Since some dog owners are afraid of aggressive confrontations with other dogs [27,70], the walking and exercise behavior of owners of dogs that exhibit aggressive behavior may be different. Thus, puppies from a breeder and adult dogs from a shelter or rescue organization might differ in the care they require and receive, including PA.

The follow-up time of the current study is significantly longer than in the earlier investigations of Serpell [38], Cutt et al. [39], Potter et al. [41] and Powell et al. [40]. The maximum follow-up period was ten months in the study of Serpell [38]. However, the results of the current study show that an observation period of several months might not be sufficient to document the total change in PA, especially when acquiring young dogs.

Earlier studies demonstrated a relationship between the size and energy level of a dog and the PA of the dog owners [24,25,26,27,30,31,32,34,35,70]. The current study found no differences in PA based on the dogs’ body size and energy levels between participants at baseline. This might suggest a causal relationship between these dog-specific variates and increased PA in owners of different dog breeds. This effect might only manifest after acquiring a dog of a certain breed. However, due to the small sample size, this could not be tested.

Earlier studies have shown that the total amounts of dog walking differ in various countries outside of Europe [71,72,73]. A prior meta-analysis states that dog owners walk their dogs on average for 46–300 min per week [74]. However, all studies taken into account in this meta-analysis were performed in North America or Japan [74]. Crozet et al. [75] speculate that there are differences in pet management practices around the world. The amount of dog-related PA in the current study is in line with earlier findings from Germany [15,49], the Netherlands [76] and France [75]. Thus, dog owners from Middle and Western Europe might differ systematically in their dog walking practices from dog owners in other parts of the world.

The fact that PA in this study exceeds earlier results by Hielscher et al. [15,49] after 24 months could be due to the fact that, in this longitudinal study, the acquired dogs were at the same young age and at the peak of their physical performance. Cross-sectional studies are likely to include dog owners with dogs of widely different ages. Since the age of the dog is negatively related to PA in dog owners [24,25,26,27,28], the mean in a sample of dog owners with young dogs is expected to be higher than in a mixed population that contains dogs of different ages. However, since very young dogs (<1 year) are more likely to be walked [25], this could lead to the conclusion that the dog owners’ PA would first increase and then decrease again after 12 months. This was not found to be the case. There is some evidence that vigorous exercise might be harmful to young dogs [42,43]. This may lead dog owners to believe that young dogs should not be exercised as much as adult dogs. Furthermore, it has been reported that puppies sleep more during the daytime than young adult dogs [29]. Thus, the American Veterinary Medical Association and the Swiss Veterinary Association for Behavioral Medicine recommend starting with short, but frequent dog walks [44,45]. These short walks can be increased over time, leading to a higher weakly duration of dog walking and thus an increase in total PA and dog-related PA [45]. This could lead to a steady increase in dog-related PA that would peak after 24 months when the dogs were considered to be fully grown.

The decrease in PA after 36 months was unexpected and might have been the result of the COVID-19 pandemic. Although dog walking was restricted during the lockdown in some countries (see e.g., [77,78]), this was not the case in Germany [79]. In the UK, dogs were walked less frequently and for shorter durations during COVID-19 lockdowns [80], and comparable results have been reported in Spain [77] and Serbia [78]. A similar phenomenon may have occurred in Germany despite the absence of restrictions, but it is difficult to draw direct comparisons due to the differences in measurement systems used in the studies. It is also possible that the novelty effect of being a dog owner wears off after 2–3 years of ownership, resulting in owners not walking their dogs as much as before. This effect might be reflected to a certain degree in the studies of Potter et al. and Powell et al. [40,41]. Both studies found a small decrease in PA when comparing the time points of 3 vs. 8 months [40] and 6 vs. 12 weeks [41] after the acquisition of the dog.

Other previous investigations that did not focus specifically on dog-related PA, have shown that the duration of PA drops significantly by about 33% to 42% during home confinement [81,82]. In comparison, the decreases in the current study are considerably smaller and the largest decrease from 24 to 36 months was found in non-dog-related PA (31.2%). Unfortunately, the current study did not ask whether there was a lockdown after 36 months when the survey was conducted and how severely these regulations affected the personal life of the participants. It is, therefore, conceivable that some participants were more restricted in their daily lives than others at the time of the survey. If this were the case, the results might suggest that the ownership of a young or middle-aged dog could serve to prevent decreases in PA resulting from the pandemic.

The question of how much dog-related PA can be considered health enhancing is still a subject of debate. Some studies suggest that at least some dog-related PA are of moderate intensity [8,83] although, Hielscher et al. [49] found that only a small part of dog-related PA fell in this category. They also state, however, that dog-related PA other than dog walking might qualify as health-enhancing PA [49]. Evidence suggests that light PA also improves health [3,84,85,86,87], albeit it is not as effective as activities at higher intensities [87,88].

Over the duration of the study period, all subscales of PA increased, except for non-dog-related PA. Given that the replacement of sitting time with light or moderate to vigorous PA is associated with lower mortality risk, especially in previously sedentary populations [89], it may be assumed that the effects found in this study are clinically relevant.

In the present work, it appears that the participants were already very active before acquiring a puppy. In the validation study of the BSA-F, Fuchs et al. report much lower levels of PA [47]. Therefore, it could also be assumed that future dog owners were already more active than a comparable group without dogs. Still, participants in this study became even more active after acquiring a dog. Consequently, the results show that especially future dog owners who have previously maintained a sedentary lifestyle and become physically active because of their dog can benefit from the acquisition of a dog and the increased level of PA associated with dog acquisition.

This study has several limitations. The small sample size reduces the power of the study and increases the margin of error. Given that breeders of 996 litters were contacted and only eleven participants completed the study, the study is very likely to suffer from self-selection bias. Therefore, it is not possible to generalize from the results. Future studies should involve the breed associations directly as a way to recruit more participants. Moreover, the study relied on self-reported measures of PA. Overreporting of PA is a well-known phenomenon in PA questionnaires [90,91,92]. Thus, it cannot be excluded that participants were not as active as they reported. However, given that the results are similar to those of earlier studies, it is likely that the questionnaire is robust and reliable. Furthermore, the study had a high dropout rate. This can be attributed to the long follow-up period. However, an earlier study with a similar follow-up period reported a dropout rate that was similar [48]. Other studies suggest that the attrition rate in the current study is in line with other health-related studies [93]. Another limitation could be social desirability in self-reported PA. Social desirability has been shown to correlate with overreporting of PA [94,95]. Although PA questionnaires are not appropriate for measuring an individual’s PA, they are stable enough to measure changes in PA in groups [90,96]. Therefore, it is necessary to replicate the current study by using objective measures, such as accelerometers or pedometers. Finally, this study is a single-arm study. Thus, it is not possible to compare it to participants who did not acquire a dog. A future replication study should either include a control group of non-dog owners or compare owners of different dog breeds against each other. The latter was planned for this study but could not be carried out due to the low rate of participant recruitment.

To our best knowledge, this is the first study to focus on the specific effects of puppy acquisition on the owner’s PA. Further, no previous study has used such a long follow-up period and thus been able to give information over such a long time period.

## 5. Conclusions

The results of the current study show, that PA increases in German dog owners within the first two years after puppy acquisition. The findings also indicate that owners’ PA remained above baseline levels during the third year of the study, despite the advent of COVID-19 pandemic-associated limitations on PA. The study provides evidence that puppy acquisition may contribute significantly to PA in prospective dog owners in Germany. Additional dog-related PA, especially dog walking, seems to be beneficial in terms of enhanced total PA. Participants did not differ at the time of dog acquisition in sociodemographic variables and PA behavior depending on the size or the energy level of their chosen dog breeds. It is, therefore, possible that differences in the PA of dog owners in earlier studies might be attributed to the size and energy level of the dogs, and that dog owners do not initially choose an animal that corresponds to their level of PA. Although this study is a pilot investigation and further research is needed, dog acquisition may represent an innovative health care intervention by lowering mortality risk and rates of several chronic diseases due to improvements in PA.

## Figures and Tables

**Table 1:** Sociodemographic variables and units.

Variable	Unit
Age of the dog owner	Years
Height of the dog owner	Meter (m)
Body weight of the dog owner	Kilogram (kg)
Body mass index (BMI) as calculated by self-reported height and weight	kg/m^2^
Gender of the dog owner	Male Female
Smoking status	Yes No
Highest educational attainment	No degree Secondary modern school qualification Intermediate high school certificate University of applied science qualification or high school diploma College or university degree Dissertation
Relationship status	In a relationship Not in a relationship
Employment status	Full time job Part time job No job
Income in €	<1000 1000–1999 2000–2999 3000–3999 4000–4999 5000–5999 6000–6999 7000–7999 8000–8999 ≥9000 Prefer not to say.
Size of hometown by number of inhabitants	<5000 5000–19,999 20,000–99,999 100,000–499,999 ≥500,000
Yard ownership	Yes No
Existence of any chronic diseases in the dog owner	Yes No
Number of dogs owned at baseline	Numeric number
Dog ownership at baseline	Yes No
Whether participants have ever owned a dog	Yes No
Time since they owned their last dog	In years

**Table 2:** Sociodemographic data at baseline for the total sample and completers only.

Variable	Category	Total Sample (*n* = 38)		Completers Only (*n* = 11)	
		*n*	%	*n*	%
Gender	Male	9	24.3	3	27.3
	Female	28	75.7	8	72.7
Smoking status	Smoker	7	18.9	3	27.3
	Non-smoker	30	81.1	8	72.7
Relationship status	Single	4	10.8	3	27.3
	In relationship	33	89.2	8	72.7
Size of hometown as measured in number of people living in the city	<5000	17	45.9	7	63.6
	5000–19,999	10	27.0	3	27.3
	20,000–99,999	4	10.8	1	9.1
	100,000–499,999	2	5.4	0	0.0
	≥500,000	4	10.8	0	0.0
Garden ownership	Yes	36	97.3	11	100.0
	No	1	2.7	0	0.0
Employment status	Employed	32	84.2	10	90.9
	Unemployed	6	15.8	1	9.1
Monthly income in €	1000–1999	3	8.3	2	20.0
	2000–2999	6	16.7	1	10.0
	3000–3999	9	25.0	4	40.0
	4000–4999	2	5.6	0	0.0
	5000–5999	5	13.9	2	20.0
	6000–6999	3	8.3	0	0.0
	7000–7999	0	0.0	0	0.0
	8000–8999	0	0.0	0	0.0
	≥9000	1	2.8	0	0.0
	No answer	7	19.4	1	10.0
Ownership of other dogs before puppy acquisition (baseline)	Yes	18	47.4	6	54.5
	No	20	52.6	5	45.5
Dog breed	Cavalier King Charles Spaniel (small size, low energy)	2	5.3	0	0.0
	West Highland White Terrier (small size, medium energy)	0	0.0	0	0.0
	Parson Russel Terrier (small size, high energy)	3	7.9	2	18.2
	Whippet (medium size, low energy)	3	7.9	1	9.1
	Labrador Retriever (medium size, medium energy)	11	28.9	2	18.2
	Border Collie (medium size, high energy)	6	15.8	1	9.1
	Bernese Mountain Dog (large size, low energy)	12	31.6	5	45.5
	Rottweiler (large size, medium energy)	0	0.0	0	0.0
	Belgian Shepherd Dog (large size, high energy)	1	2.6	0	0.0

**Table 3:** Absolute and relative changes in PA behavior over time (completers only).

		Leisure Time Walking in h/Week	Dog Walking in h/Week	Dog-Related PA in h/Week	PA without a Dog in h/Week	Total PA in h/Week
**T0**	*M* (*SD*)	6.09 (7.82)	5.70 (8.07)	6.91 (9.30)	6.96 (8.37)	13.88 (14.73)
	*Mdn* (*IQR*)	2.81 (0.00, 7.00)	1.50 (0.00, 7.00)	4.25 (0.00, 9.17)	3.25 (0.00, 12.04)	10.79 (6.42, 15.00)
	*n*	11	11	11	11	11
**T1**	*M* (*SD*)	8.38 (3.80)	8.06 (3.41)	9.84 (3.95)	7.04 (4.78)	16.88 (7.48)
	*Mdn* (*IQR*)	8.75 (5.83, 10.50)	8.75 (5.83, 10.50)	9.38 (5.83, 14.00)	6.46 (4.00, 9.38)	14.83 (11.33, 22.17)
	Δ_T0-T1_ (Δ%_T0-T1_)	2.29 (+37.60)	2.36 (+41.40)	2.93 (+42.40)	0.08 (+1.15)	3.00 (+21.61)
	*n*	11	11	11	11	11
**T2**	*M* (*SD*)	9.57 (4.87)	9.57 (4.87)	11.61 (6.56)	5.52 (5.58)	17.14 (11.35)
	*Mdn* (*IQR*)	10.42 (7.00, 14.00)	10.42 (7.00, 14.00)	11.58 (7.00, 14.63)	3.00 (1.56, 8.58)	16.75 (8.56, 22.50)
	Δ_T0-T2_ (Δ%_T0-T2_)	3.48 (+57.14)	3.87 (+67.89)	4.70 (+68.02)	−1.44 (−20.69)	3.26 (+23.49)
	*n*	11	11	11	11	11
**T3**	*M* (*SD*)	13.91 (7.81)	13.59 (7.79)	15.54 (8.56)	9.47 (9.63)	25.00 (16.56)
	*Mdn* (*IQR*)	14.00 (7.00, 21.00)	14.00 (7.00, 21.00)	14.42 (8.75, 22.00)	6.25 (2.58, 13.00)	20.75 (11.25, 35.00)
	Δ_T0-T3_ (Δ%_T0-T3_)	7.82 (+128.41)	7.89 (+138.42)	8.63 (+124.89)	2.51 (+36.06)	11.12 (+80.12)
	*n*	11	11	11	11	11
**T4**	*M* (*SD*)	10.97 (8.75)	10.34 (7.57)	11.84 (7.65)	6.52 (7.58)	18.36 (12.73)
	*Mdn* (*IQR*)	7.00 (5.25, 21.00)	7.00 (5.25, 21.00)	7.00 (6.25, 21.00)	6.02 (0.50, 10.00)	13.50 (7.50, 24.83)
	Δ_T0-T4_ (Δ%_T0-T4_)	4.88 (+80.13)	4.64 (+81.40)	4.93 (+71.35)	−0.44 (−6.32)	4.48 (+32.28)
	*n*	11	11	11	11	11
	*χ*^2^ (*df*)	11.94 (4)	11.73 (4)	10.09 (4)	3.91 (4)	11.20 (4)
	*p*	0.018	0.020	0.039	0.419	0.024
	Kendall’s *W*	0.36	0.36	0.31	0.12	0.34

*Note. IQR*, interquartile range; *M*, mean; *Mdn*, median; *SD*, standard deviation; *p*-value, changes over time within the completing population (*n* = 11) using the Friedman-test.

## Data Availability

The data are available upon request from the first author.
